# Observation of pendular butterfly Rydberg molecules

**DOI:** 10.1038/ncomms12820

**Published:** 2016-10-05

**Authors:** Thomas Niederprüm, Oliver Thomas, Tanita Eichert, Carsten Lippe, Jesús Pérez-Ríos, Chris H. Greene, Herwig Ott

**Affiliations:** 1 Research Center OPTIMAS, Technische Universität Kaiserslautern, 67663 Kaiserslautern, Germany; 2 Graduate School Materials Science in Mainz, Staudinger Weg 9, 55128 Mainz, Germany; 3 Department of Physics and Astronomy, Purdue University, West Lafayette, Indiana 47907, USA

## Abstract

Engineering molecules with a tunable bond length and defined quantum states lies at the heart of quantum chemistry. The unconventional binding mechanism of Rydberg molecules makes them a promising candidate to implement such tunable molecules. A very peculiar type of Rydberg molecules are the so-called butterfly molecules, which are bound by a shape resonance in the electron–perturber scattering. Here we report the observation of these exotic molecules and employ their exceptional properties to engineer their bond length, vibrational state, angular momentum and orientation in a small electric field. Combining the variable bond length with their giant dipole moment of several hundred Debye, we observe counter-intuitive molecules which locate the average electron position beyond the internuclear distance.

## Abstract (web summary)

Rydberg molecules have potential for ultracold chemistry applications in light of their unconventional binding mechanism that provides high tunability. Here the authors observe and control butterfly Rydberg molecules, which are bound by a shape resonance in the electron-perturber scattering.

## Graphical abstract

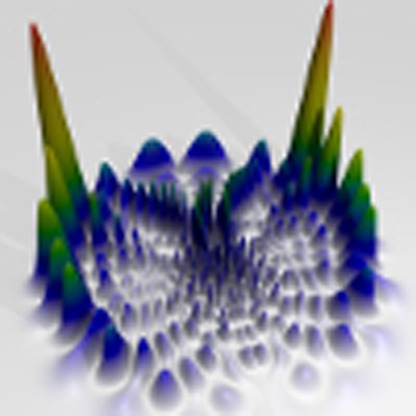

Controlling the internal and external quantum states of molecules is the central goal of ultracold chemistry^1^ and allows for the study of coherent molecular dynamics, collisions^2^,^3^,^4^,^5^ and tests of fundamental laws of physics^6^. If the molecules additionally possess a permanent electric dipole moment, the study of dipolar quantum gases and spin systems with long-range interactions^7^,^8^ as well as applications in quantum information processing^9^,^10^,^11^ are possible. Rydberg molecules constitute a class of exotic molecules, which are bound by the interaction between the Rydberg electron and a ground state atom. This peculiar interaction represents a novel chemical binding mechanism, that is based on the elastic *S*- and *P*-wave scattering between the electron in a Rydberg state and a ground state perturber atom, which is located inside the electron wavefunction.

The scattering is described by a Fermi-type contact interaction with an energy-dependent scattering length^12^,^13^,^14^,^15^. The bond length of these molecules is connected to the extension of the electron wavefunction and can reach hundreds of nanometres^14^,^16^. In the extreme case, the interaction with the ground state atom can substantially displace the electron from the ionic core, giving rise to permanent electric dipole moments in the kilo-Debye range^17^. This holds even in the case of homonuclear molecules due to the vanishing exchange energy. The extreme properties of Rydberg molecules make them versatile objects for the study of low-energy electron–atom scattering, precision calculation of potential energy curves^18^, molecular dipole–dipole interaction, Rydberg blockade and anti-blockade effects in molecular samples as well as complex molecular dynamics.

Due to the presence of a shape resonance in the *P*-wave scattering, a special type of Rydberg molecules with large binding energies and exceptional properties arises. These so-called butterfly molecules were predicted theoretically in 2002 (refs 19, 20) but clear experimental evidence for their existence was missing so far. Here, we report on the photoassociation of butterfly Rydberg molecules and their orientation in a weak electric field. Starting from a Bose–Einstein condensate of rubidium atoms, we fully control the external degrees of freedom and spectroscopically resolve the rotational structure and the emerging pendular states in an external electric field. This not only allows us to extract the bond length, the dipole moment and the angular momentum of the molecule but also to deterministically create molecules with a tunable bond length and orientation. We anticipate direct applications in quantum chemistry, many-body quantum physics and quantum information processing.

## Results

### Butterfly Rydberg molecules

**Figure 1:**
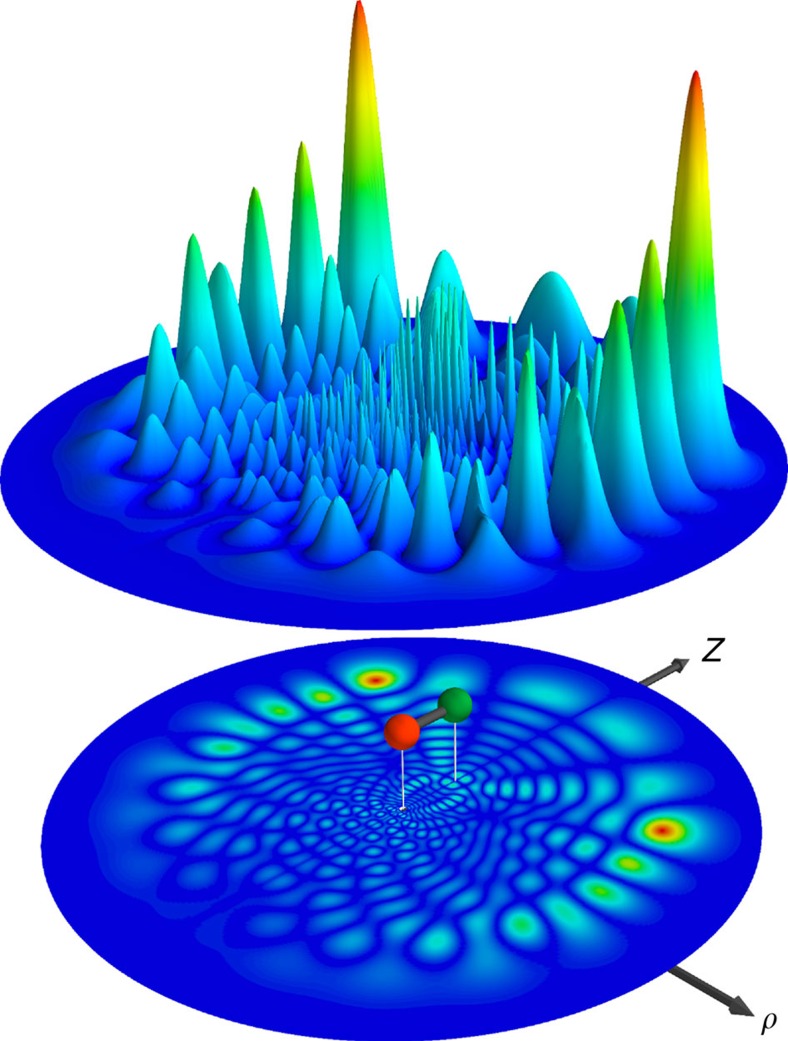
Radial electron density of a butterfly molecule. The upper plane shows a surface plot of the radial electron density *ρ*|Ψ(*z*,*ρ*)|^2^ for a butterfly molecule near the 25*p*-state of rubidium. The lower plane shows the two-dimensional projection of the electron density. A sketch of the molecule above the projection plane shows, where the Rb^+^ ion (red) and the ground state perturber (green) are located. The bond length is 245 a_0_.

**Figure 2:**
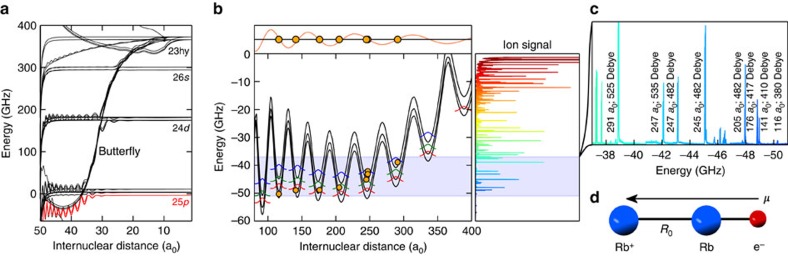
Butterfly Rydberg molecules. (**a**) Adiabatic potential energy curves (PEC) for the Rydberg ground state interaction in the vicinity of the 25*p*-state. The butterfly state detaches from the *n*=23 hydrogenic manifold and crosses the lower lying 26*s*, 24*d* and 25*p*-state. A zoomed-in inner region of the lowest butterfly potentials (red lines) is shown in (**b**). Three different PECs (black lines) emerge, corresponding to the quantum numbers 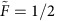, 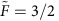 and 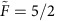 of the total spin 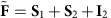. The oscillatory shape provides a set of harmonic potential wells at different bond lengths. The lowest bound states for each PEC are sketched in red, green and blue. The orange points represent the experimentally obtained values for the binding energy and the bond length of the eight molecular states studied in detail (see text). The extension of the points denotes the estimated error in the bond length of 5 a_0_. The absolute frequency uncertainty is 10 MHz (see Methods). The top panel shows how the measured bond lengths (orange points) coincide with the nodes of the 25*p*_1/2_, *m*_*j*_=1/2 radial wavefunction (light red). The right panel shows the experimental spectrum. The colour code is a guide to the eye. The zoom on the lowest energy peaks of the experimental spectrum (**c**) shows the determined bond length and the dipole moment of the respective peak. (**d**) A sketch to scale of the molecule constituents, averaged z-positions for a butterfly state with a bond length of 116 a_0_. The average position of the electron is located beyond the perturber atom.

Butterfly Rydberg molecules are dominated by the *P*-wave scattering between the Rydberg electron and the ground state atom, which maximizes the gradient of the electron wavefunction at the position of the ground state atom (see Methods). As a consequence, all angular momentum states of the Rydberg electron are mixed and the electron density acquires a characteristic density distribution which resembles a butterfly (Fig. 1). The first spectroscopic evidence for the existence of this mechanism has been reported in refs 21, 22. The calculation of the corresponding potential energy curves (PEC) in the Born–Oppenheimer approximation requires the diagonalization of the full Hamiltonian, which in our approach includes the fine structure in the Rydberg atom, the hyperfine interaction in the perturber atom, and all *S*- and *P*-wave scattering processes (see Methods). The PECs in the vicinity of the 25*p*-state are shown in Fig. 2a. The butterfly states detach from the *n*=23 hydrogenic manifold and cross all lower-lying states down to the 25*p*-state. The red PECs are relevant for this work and the bound states in the strongly oscillating part of the PEC are the butterfly molecules under investigation. Even for the deepest potential wells the electron wave functions in these PECs possess a 15% admixture of the 25*p*-state, which we use to excite the butterfly molecules with a single photon transition.

### Photoassociation spectroscopy

The experiment was performed in a Bose–Einstein condensate (BEC) of ^87^Rb with a peak particle density of 4 × 10^14^ cm^−3^, an atom number of 2 × 10^5^ and a temperature of 100 nK. All spin states of the 5*s*_1/2_, *F*=1 ground state were populated. The photoassociation of the butterfly molecules was achieved using a single photon transition to the energetic vicinity of the adiabatic free two-particle state 25*p*_1/2_⊗5*s*_1/2_, *F*=1, which serves as reference point for our spectroscopy throughout this work. Since the created butterfly molecules can decay into ions by photoionization and associative ionization^23^, the produced ions are used as a probe for the creation of the molecule.

The experimental sequence consists of 500 ms continuous excitation and ion detection. By detuning the excitation laser up to −60 GHz in steps of 2 MHz we obtain the spectrum shown to the right of Fig. 2b. As the laser detuning is gradually increased, we start to probe the bound states in the butterfly potential. We observe a plenitude of molecular states up to an energy of −50 GHz. This is in accordance with the calculated PECs. Moreover, the density of molecular states drops at detunings below −40 GHz, which marks the transition to a spectral region, where only the lowest bound states in each potential well are populated. The spectroscopic results directly prove the existence of butterfly molecules.

### Pendular states

We now focus on the ground state molecules in each potential well and demonstrate, how butterfly molecules with selected bond length and high degree of orientation can be created. Due to the symmetry breaking caused by the localized valence electron of the perturber, butterfly molecules, along with the related class of so-called trilobite molecules, are the only known homonuclear molecules with a large permanent electric dipole moment^14^,^15^,^17^,^24^. The interaction of the dipole moment ***μ*** with an external electric field **F** significantly changes the rotational structure of the molecule. In the simplest approximate description of a dipolar rigid rotor that neglects the coupling of the nuclear motion to the electronic and nuclear spin degrees of freedom, the eigenstates of energy *E* need to fulfil (see Methods)


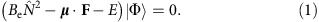


**Figure 3:**
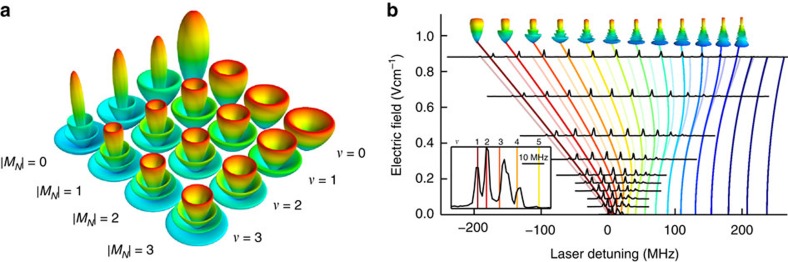
Pendular states. (**a**) Plot of the orbitals |Φ(*θ*,*φ*)| for pendular states |*ν*,*M*_*N*_〉 of a dipolar molecule with *μ*=482 Debye in an electric field of 1 V cm^−1^. (**b**) Spectroscopy of the butterfly state at −47.9 GHz in different electric fields. Each spectrum is normalized and shifted on the *y* axis according to the applied electric field. The coloured lines show the eigenenergies of a rigid rotor with a permanent dipole moment in an electric field (equation (1)) for |*M*_*N*_|=0 (weak lines) and |*M*_*N*_|=1 (strong lines). The dipole moment and the bond length are adapted to fit the experimental data. In this case, we find *R*_0_=205 a_0_ and *μ*=482 Debye. The inset shows a zoom of the low-field (≈30 mV cm^−1^) measurement together with the calculated position of the first five eigenstates of the dipolar rotor model with |*M*_*N*_|=1. We attribute the appearance of higher *ν* states at field strength *F*=0 to the mixing of higher *N* states in the residual field (see Methods).

In equation (1), the zero of the energy axis is chosen to coincide with the rotationless vibrational energy level being studied. For vanishing electric field, the molecule behaves as a rigid rotor of length *R*_0_ and reduced mass *m*_r_=*m*_Rb_/2 using the rotational quantum number *N*, its projection on the molecular axis *M*_*N*_ and the rotational constant *B*_e_=*ℏ*^2^/2*I*, where *I*=*m*_r_*R*_0_^2^ represents the moment of inertia of the rigid rotor. When, on the other hand, the interaction with the electric field dominates the rotational constant 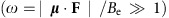, the molecule enters so-called pendular states^25^,^26^, which are characterized by the quantum number *ν* and differ in the degree of orientation of the molecule with respect to the field axis (Fig. 3a). In this regime only the angular momentum projection *M*_*N*_ remains a good quantum number, while the rotational quantum numbers *N* are strongly mixed.

In Fig. 3b, we show the electric field-dependent spectra of the butterfly state at −47.9 GHz along with the fitted theory of equation (1). Due to the huge dipole moments of the butterfly molecules, fields of 1 V cm^−1^ are sufficient to put the system deep in the pendular regime. Varying the electric field, we can directly observe the crossover from rotational states to pendular states. At fields of 4 V cm^−1^, the degree of orientation *ω* exceeds previous record values^26^ by a factor of two. Since we photoassociate the butterfly molecules in a Bose–Einstein condensate, which has no angular momentum in the centre of mass motion, only states with |*M*_*N*_|=0 and |*M*_*N*_|=1 can be observed (see Methods). This leads to a very clean spectrum of pendular states, which, in contrast to previous studies^16^,^17^,^21^,^22^,^24^, allows to determine the dipole moment *μ* with an estimated precision of 10 Debye and the bond length *R*_0_ with an estimated precision of 5 a_0_.

Applying this method to eight of the lowest spectroscopic lines in the butterfly spectrum, we can extract the respective dipole moments and bond lengths (Fig. 2c). This additional information enables an improved interpretation of the butterfly spectrum and comparison with theory. This is demonstrated in Fig. 2b, where the orange points mark the measured bond lengths and binding energies for the eight studied lines. The energetic deviation of the measured points from the predicted bound states is only few per cent of the binding energy and is most likely explained by inaccurate *P*-wave scattering phase shifts assumed in the model. It is remarkable how well the measured bond lengths agree with the wells of the model potential. In fact, the positions of the wells are mainly determined by the strong gradient at the radial nodes of the *p*-state electronic wavefunction and thus the bond length of the butterfly molecules is an indirect measure for the position of the 25*p* radial nodes. A comparison of the measured bond lengths with the nodes of the calculated 25*p*_1/2_ wavefunction is shown in the top panel of Fig. 2b.

### Properties

Butterfly molecules also exhibit counter-intuitive properties. In a semiclassical picture of molecules with one valence electron, the average position of the electron is always located between the two nuclei. The maximum possible dipole moment is thus limited by the bond length, as realized, for example, in an ionic bond. The strong interference of the electron caused by the scattering with the ground state atom (Fig. 1) permits the butterfly molecules to exceed this limit and locate the electron's average position beyond the perturber (Fig. 2d). This effect is seen experimentally for the deepest bound butterfly molecules. The state at −50.3 GHz possesses a dipole moment of 380 Debye, which is 30% larger than that of two opposite charges separated by the measured bond length of 116 a_0_. This phenomenon is even stronger in the theoretical model, which reveals a dipole moment of 442 Debye for the studied state. Throughout all investigated butterfly states the measured dipole moments are systematically lower than the calculated values but do not deviate by >35%.

Due to the deep potential wells (Fig. 2b), inward tunnelling and subsequent decay should not limit the lifetime of the studied butterfly molecules. In a separate experiment using short excitation pulses (see Methods) we were able to determine the lifetimes of the strongest observed butterfly molecules. In the high-density environment of a BEC we find a lifetime of 5 μs which is mainly limited by inelastic collisions with additional ground state atoms. Extrapolating to zero ground state density to mitigate the effect of collisions we find a lifetime on the order of 20 μs, which is compatible with the lifetime of the 25*p* Rydberg state.

## Discussion

The long lifetime along with the negligible motion of the ground state atoms in the BEC enables narrow photoassociation lines and thus a clean optical spectroscopy. This allows for the deterministic creation of vibrational ground state molecules with a selected bond length and dipole moment. In a weak electric field we can furthermore restrict the rotation of the molecule and precisely set the orientation of the molecule with respect to the field axis. This high degree of control over molecules with giant dipole moments even in a sample with only one atomic species opens many perspectives for future applications. The high precision of the molecular spectroscopy can serve as a benchmark for quantum chemical calculations of molecular states. Rydberg molecules are promising candidates to implement dipolar interactions in many-body quantum systems. The comparable energy scale of the rotational structure and the dipole–dipole interaction is unique in the world of polar molecules and will complement studies using heteronuclear alkali dimers in the rovibrational ground state^7^. Starting from pre-associated weakly bound ground state molecules in a three-dimensional optical lattice, spin physics with pendular states are feasible^7^. Dressing weakly bound molecules with a Rydberg molecule might even allow to study coherent tunnelling dynamics with long-range interactions. It will be interesting in the future to extend the concepts of the Rydberg blockade and anti-blockade to interacting Rydberg molecules. Butterfly molecules might also be a good starting point to prepare heavy Rydberg systems^27^. Ultimately pendular states of dipolar molecules could also be a building block to scalable quantum information processing^9^,^11^.

## Methods

### Calculation of the butterfly PECs

Rich spectral features, such as average line shifts and collisional broadening, arise when a Rydberg atom is excited in a high-density gas. These effects have been understood since Fermi's idea of a quasi-free electron^12^ could successfully explain the early experiments by Amaldi and Segrè^28^. Extending Fermi's work, Omont later generalized the zero-range *S*-wave potential to higher partial waves^13^. The contribution of the *P*-wave becomes particularly important in alkali atoms, as those show a low-energy shape resonance in the ^3^*P*^o^ scattering channel. To model the high-resolution spectroscopy of rovibrational diatomic Rydberg levels, we not only include the singlet electron–Rb *S*-wave and *P*-wave scattering information but also the spin–orbit splitting of the Rydberg state and the ground state ^87^Rb(5*s*_1/2_) hyperfine structure. Thus, the Hamiltonian for the Rydberg–perturber interaction in atomic units reads as^29^


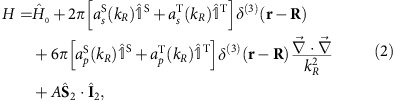


where the position of the Rydberg electron is denoted as **r**, whereas the position of the perturber from the Rydberg core is expressed as **R**. 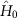 represents the unperturbed Rydberg states involved in the calculations, accounting for the appropriate quantum defects^30^. *a*_*s*_^S^(*k*_*R*_) and *a*_*s*_^T^(*k*_*R*_) denote the singlet and triplet *S*-wave *e*^−^–Rb(5*S*) scattering lengths, and analogously *a*_*p*_^S^(*k*_*R*_) and *a*_*p*_^T^(*k*_*R*_) represent the singlet and triplet *P*-wave *e*^−^–Rb(5*S*) scattering lengths, respectively^15^. The momentum of the Rydberg electron evaluated at the perturber position is 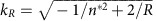, where n* stands for the effective quantum number of the level of interest. In equation (2), the triplet and singlet components of the *e*^−^–Rb(5*S*) scattering lengths are associated with the projectors 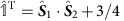 and 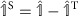, respectively. The last term in equation (2) represents the hyperfine structure of the perturbing atom ^87^Rb 5*s*_1/2_ with *A*=3.417 GHz. The present Hamiltonian is diagonalized in a truncated basis of atomic levels, along with the electron and nuclear spin degrees of freedom for the perturbing ground state atom, that is, 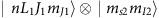, where *L*_1_, *J*_1_ and *m*_*J*1_ are associated with the Rydberg electron as in refs 29, 31, whereas |*m*_*s*2_*m*_*I*2_〉 stands for the hyperfine levels in the perturbing atom.

### Dipolar rigid rotor model

The simplest approximate description for the observed rotational structure of the Rydberg molecules includes only the squared rotational angular momentum operator 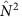, and thus ignores coupling to the electronic and nuclear spin degrees of freedom. Due to the particular nature of the butterfly potentials also the mixing of vibrational states can be neglected. The otherwise vanishing overlap of the nuclear wavefunction allows only for states located in the same well to be mixed. Since the spacing of the vibrational levels inside the wells is higher than 3.5 GHz while the splitting of the rotational states in the small applied fields is only 300 MHz we do not expect significant mixing of the vibrational states in the field. However, for higher electric field strength this mixing has to be taken into account.

### Experimental setup

Starting from a three-dimensional magneto-optical trap, a Bose–Einstein condensate of ^87^Rb is prepared by forced evaporation in a crossed YAG dipole trap. We reach a BEC of 2 × 10^5^ rubidium-87 atoms at final trapping frequencies of 2*π* × 150 Hz in the radial and 2*π* × 80 Hz in the axial direction. The photoassociation of the butterfly molecules is done with a frequency doubled, continuous wave dye laser at a wavelength of 297 nm and a total power of 20 mW, focused to a beam waist of 40 μm. The excitation pulse has a duration of 500 ms, during which the generated ions are detected with a discrete dynode electron multiplier. A small electric field of 30 mV cm^−1^ at the position of the BEC is needed to guide the ions to the detector and is thus always present, if not higher fields are applied. The total detection efficiency for a single created ion is 44%. For the measurements presented here the linear polarization of the excitation light was orthogonal to the electric field axis, yielding strong transitions to the *M*_*N*_=1 states (see also Methods). The full experimental spectrum (Fig. 2b) consists of multiple individual measurements spanning up to 2 GHz, which is the maximum continuous detuning range of the employed transfer cavity lock. To combine the individual measurements we use the absolute frequency reference of a Fizeau-interferometer-based wavemeter. The uncertainty in the observed binding energies are thus mainly limited by the wavemeter's resolution of 10 MHz.

The lifetime of the butterfly molecules was measured in a time of flight experiment, as described in ref. 23. After a short laser pulse of 1 μs the produced atomic and molecular ions are recorded. We extract the lifetime from the exponential decay of the molecular ion signal.

### Polarization dependence

**Figure 4:**
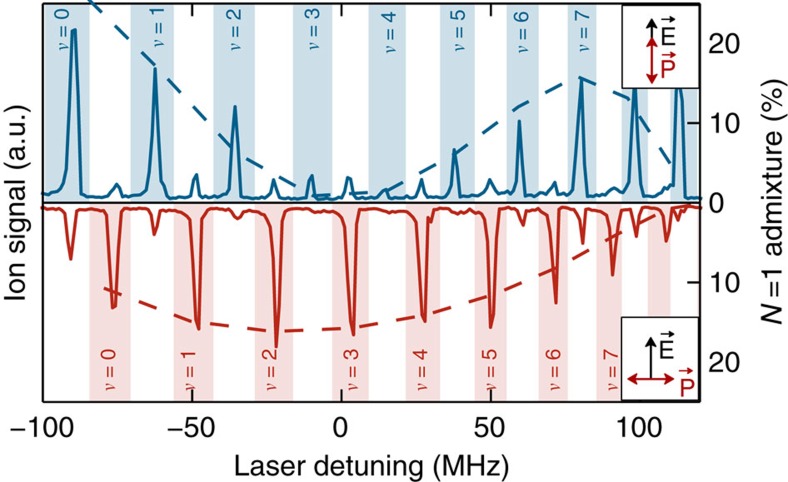
Polarization dependent coupling strength of the pendular states. The spectrum of the butterfly state at −47.9 GHz in an electric field of 0.44 V cm^−1^ is shown for two different angles between the linear laser polarization and the electric field axis. While for parallel polarization (solid blue line) mainly |*M*_*N*_|=0 states (blue patches) are excited, we see that for orthogonal polarization (solid red line) mainly |*M*_*N*_|=1 states (red patches) are excited. In both cases the qualitative behaviour is nicely reproduced by the admixture of the *N*=1 state to the corresponding pendular state that is extracted from the the rigid rotor model (dashed lines).

Since we photoassociate butterfly molecules from a BEC that has no angular momentum in the centre of mass motion, only rotational states with *M*_*N*_=0 and *M*_*N*_=±1, corresponding to *π* and *σ*^±^ transitions, respectively, can be excited. A comparison of the pendular state spectroscopy for different orientation of the linear laser polarization is presented in Fig. 4. If the laser polarization is parallel to the electric field (blue) we mainly drive a *π* transition and the |*M*_*N*_|=0 lines dominate the spectrum. If, on the other hand, the laser polarization is orthogonal to the electric field axis (red), we drive both *σ*^+^ and *σ*^−^ transitions and thus mainly couple to the |*M*_*N*_|=1 states.

Since the BEC provides an initial state with *N*=0, *M*_*N*_=0 and the usual angular momentum selection rules Δ*N*=0,±1 (but *N*=0→*N*'=0 forbidden) need to be fulfilled in the photoassociation process, we can only couple to |*N*=1, *M*_*N*_=0,±1〉 states. The coupling strength is therefore proportional to the admixture of the *N*=1 state (Fig. 4).

### Data availability

The data that support the findings of this study are available from the corresponding author upon request.

## Additional information

**How to cite this article:** Niederprüm, T. *et al*. Observation of pendular butterfly Rydberg molecules. *Nat. Commun.* 7:12820 doi: 10.1038/ncomms12820 (2016).
